# Depression as a risk factor for Alzheimer’s disease: A human post-mortem study

**DOI:** 10.1371/journal.pone.0320561

**Published:** 2025-04-03

**Authors:** Mizuki Morisaki, Farnoosh Rezaali, Laurie C. Lau, Delphine Boche, Golam M. Khandaker, Gustavo Turecki, Lindsey I. Sinclair

**Affiliations:** 1 Dementia Research Group, University of Bristol, Bristol, United Kingdom; 2 Clinical and Experimental Sciences, Faculty of Medicine, Sir Henry Wellcome Laboratories, University of Southampton, Southampton, United Kingdom; 3 Clinical Neurosciences, Clinical and Experimental Sciences, Faculty of Medicine, University of Southampton, Southampton, United Kingdom; 4 MRC Integrative Epidemiology Unit, University of Bristol, Bristol, United Kingdom; 5 Centre for Academic Mental Health, Population Health Sciences, Bristol Medical School, Bristol, United Kingdom; 6 NIHR Bristol Biomedical Research Centre, Bristol, United Kingdom; 7 Douglas Bell Brain Bank, McGill University, Montreal, Canada; Taipei Veterans General Hospital, Taiwan

## Abstract

Depression is associated with persistent low mood. In mid to late life, it has been identified as a risk factor for Alzheimer’s disease (AD) with evidence that depression might be an early manifestation of AD. Although the underlying mechanisms by which depression enhances AD development remain unknown, there are several features commonly seen in both diseases such as the presence of neuroinflammation. In this study, we aimed to identify whether neuroinflammation is increased in depression as observed in the early stages of AD by examining post-mortem human brain tissue. Post-mortem human brain tissue from 54 cases with depression and 37 controls without depression were retrieved from the Douglas Bell Canada Brain Bank. Sixteen early-stage AD cases defined as a Braak stage III-IV and 15 controls were sourced from the South West Dementia Brain Bank. Frozen tissue from the dorsal prefrontal cortex was obtained for all cases in order to measure inflammatory proteins (IFN-γ, IL-1β, IL-2, IL-4, IL-6, IL-8, IL-10, IL-12p70, IL-13, and TNF-α) and endothelial markers (ICAM-1, VCAM) using ELISA and MesoScale Multiplex Assays. In the depression group, increase of IL-6 and IL-10, and decrease of IL-1β were observed compared to controls, with no changes detected for the other cytokines and the endothelial markers. In early-stage AD cases, only increased ICAM-1 expression was found compared to controls, indicating endothelial activation as an early feature of AD. None of the cytokines measured showed alteration of their expression in early-stage AD cases. Depression, but not AD, was associated with evidence of neuroinflammation. Depression may increase AD risk through different mechanism(s) than inflammation.

## Introduction

Depression (most commonly known as major depressive disorder: MDD) is a mental disorder associated with continuous low mood and loss of pleasure [1,2]. MDD is common throughout life (i.e., lifetime prevalence 4.4% - 20% [3,4]), but mid to late life MDD has been specifically identified as a risk factor for developing dementia later in life [5–7]. Individuals who have had MDD in mid to late life are twice as much as likely to develop dementia [7]. Occurrence of MDD is also common after diagnosis of dementia and comorbidity with dementia is estimated to be 30% [8,9]. Depression in mid to later life has been hence hypothesised to be an early manifestation of dementia [10], but the exact mechanism by which MDD increases risk of developing dementia remains unknown. Despite the fact that depression is a risk factor for AD, our previous study showed no evidence of increased Aβ/tau in the brain tissue from patients with depression in either early (18–50 years old) and late (51–90 years old) stage of life [11], suggesting that depression may increase risk of AD through other factors.

Increased peripheral inflammatory response has been associated with both depression and dementia [12–15]. However, cytokine expression in peripheral fluids such as serum can be affected by many different environmental factors [16] and may not fully reflect the neuroinflammatory status present in the central nervous system (CNS). This study took a novel approach using human post-mortem brain tissue from the region particularly affected by depression (i.e., dorsolateral prefrontal cortex (DLPFC)) [17–19] to investigate whether depression mirrors the neuroinflammatory status found in the early stages of Alzheimer’s disease (AD). Depression is a known factor to increase the risk of AD [20], and neuroinflammation could be one of the factors that drives AD disease development.

Alzheimer’s disease (AD) is the most common form of dementia accounting for 60 – 70% of all cases [21]. AD shares several biological features with MDD; including shared genetic variation [22,23], structural changes in the hippocampus [24], and increased inflammatory response [13,25]. Increased peripheral inflammation has been reported in both diseases and studies have suggested that chronic inflammation may play a role in their respective pathophysiology [26–28]. In MDD, prolonged activation of immune system [29–31] was identified with increased pro-inflammatory markers such as cytokines (interleukin (IL)-1, IL-2, IL-6, IL-8, IL-12, interferon (IFN)-γ, tumour necrosis factor (TNF)α) and adhesion molecules (intercellular molecule (ICAM)-1), while anti-inflammatory markers (e.g., IL-10) were reduced [32–34]. Additionally, a meta-analysis evaluating 438 MDD cases and 350 non-MDD cases reported significant elevation of TNFα and IL-6 in MDD [4], consistent with another systematic review showing increased TNFα and IL-6 in MDD, suggesting an overall elevation of peripheral inflammation [35]. However, this systematic review also reported an increase of anti-inflammatory cytokine IL-10 [35], highlighting the complexity of MDD as a condition associated with a dysregulated immune system[35]. Of note, MDD often co-occurs with other inflammatory diseases such as inflammatory bowel disease where it has been reported that 44.4% of patients with inflammatory bowel disease experienced depression [36].

Chronic inflammation is a well characterised phenomenon of AD [12,25]. Dysregulated inflammatory response caused by imbalanced pro- and anti-inflammatory cytokines is key to AD pathology. Some authors argue that increased inflammation could be detected years before the AD diagnosis [37], with studies reporting increased inflammation associated with the onset of AD. For example, proinflammatory cytokine IL-1β in serum was significantly elevated in patients with mild cognitive impairment (MCI) compared to controls [38]. Moreover, patients aged > 65 years revealed increased serum IL-1β and TNFα in people who developed AD after 2 years of follow up [17]. These results suggest inflammation is associated with AD, particularly at its early stages.

Neuroinflammation is a recognised hallmark of AD pathology [12,39–41], but whether dysregulation of the immune system is the common mechanism by which MDD could contribute to increase the risk of AD remains unknown. Furthermore, limited knowledge is available regarding neuroinflammation in MDD as most studies only examined peripheral inflammatory markers.

In this study, we explored whether neuroinflammation and downstream vascular markers of increased inflammation are increased in MDD and in the early stages of AD using post-mortem human brain tissue from individuals with MDD or AD prior to their demise compared to age- and sex-matched controls. We hypothesise that individuals with MDD will show a similar increase of neuroinflammation to that seen in early-stage AD. We focused on inflammation markers in the human post-mortem brain tissue rather than microglia (main immune cells in the brain) itself as our previous study showed no difference between MDD and AD groups [42].

## Materials and methods

### Cases

Human brain tissue was obtained from the South West Dementia Brain Bank (Bristol, UK) and the Douglas Bell Brain Bank (Montreal, Canada). Controls brains were defined as non-neurological non-neuropathological normal brains and were age, gender and post-mortem delay matched as far as possible with the disease groups. The depression cohort was composed from 54 cases with depression and 37 controls who did not suffer with depression. The early-stage AD cohort consisted of 16 early-stage AD cases based on a Braak stage of III/IV and 15 controls (See Table 1 for more details). pH values are presented in Table 1 to ensure the quality of tissue is adequate for further analysis. The data was first accessed for research purposes on 30^th^ March 2018. The data was anonymised at all time, but it contains potentially identifiable clinical information on rare events (i.e., suicide).

**Table 1:** Demographic and clinical data of the cases.

	Early-stage AD n = 16	Early-stage AD controls n = 15	MDD n = 54	MDD Controls n = 37	Statistical analysis
Female, Male (%)	25,75	40,60	35,65	22,78	Chi^2^ test p = 0.42
Age at death (years) Mean (SD)	85 (5.9)	83.3 (6.3)	49.6 (17.9)	49.1 (19.1)	Kwallis Χ^2^ = 59.36 p < 0.001***
Post-mortem delay (hour) Mean (SD)	43.6 (34.3)	52.2 (23.8)	53.1 (23.0)	47.6 (28.1)	Kwallis Χ^2^ = 4.16 p = 0.25
pH, Mean (SD)	N/A^+^	6.41 (0.22)	6.57 (0.33)	6.46 (0.33)	N/A
Cause of death (%)					Chi^2^ test p < 0.001***
Suicide	0	0	100	0	
Accident	0	33	0	34	
Natural causes	0	20	0	66	
Others/data unavailable	100	47	0	0	
Method of suicide (%)	N/A	N/A		N/A	N/A
Hanging			63		
Jumping						14.8
Intoxication			16.7		
Other			5.6		
			DSM Axis 1 Diagnosis (%)							
Nil	0	53	0	100	N/A
Major depressive disorder	0	0	78*	0	
Depressive disorder NOS	0	0	22*	0	
Dementia	100	0	0	0	
Data unavailable	0	47	0	0	
Braak stage (%)		N/A	N/A	N/A	N/A
III	13				
IV	50				
N/A	38				
Secondary histopathological analysis for dementia cases (%)^#^		N/A^+^	N/A	N/A	N/A
Early-stage AD	44				
Possible AD	13				
Probable AD	25				
Definite AD	19				
Antidepressant medication in last 3 months (%)					Chi^2^ test p < 0.001***
No	0	40	46	84	
Yes	0	0	43	5	
Unknown/others	100	53	4	8	
Data unavailable	0	7	7	3	
Antipsychotic medication in last 3 months (%)					Chi^2^ test p < 0.001***
No	0	40	40	81	
Yes	0	7	26	0	
Unknown/others	100	50	6	9	
Data unavailable	0	3	28	10	
Marital status (%)	N/A	N/A			N/A
Single			20	35	
Married/Common law			32	22	
Divorced/Separated/Widow(er)			32	14	
Dating partner			9	14	
N/A			1	0	
Data unavailable			6	15	
Educational level (%)	N/A	N/A			N/A
No high school diploma			43	43	
High school diploma or equivalent			35	30	
College diploma			7	5	
Bachelor			7	14	
Graduate (Master/PhD)			7	3	
Data unavailable			1	5	

+ N/A: not applicable, ++ SD: standard deviation, and

# confirmed to be Alzheimer’s disease. * in this study, MDD is used as an umbrella term including both major depressive disorders and depressive disorder NOS.

For the depression and control cohorts, detailed information on psychiatric illness ante-mortem was available, as the Douglas Bell Brain Bank performed detailed interviews with relatives of the donor at a suitable time after brain donation which is known as psychological autopsies [43]. Criteria retained within the MDD group include: individuals with depression meeting DSM-IV/ DSM-V criteria either at the time of death or in the 2 years preceding their demise. Individuals were excluded if they had evidence of a dementing illness either ante-mortem or on post-mortem examination, or major neuropathological abnormalities (e.g., cancer), or evidence of a widespread neurological disease process other than those under investigation (e.g., multiple sclerosis). While information was available as whether individuals had been taking antidepressants, this was not the case regarding the anti-inflammatory medication. A full description of this cohort has been previously published [44].

In the early-stage AD group, individuals had to have a diagnosis of MCI/AD made in the 2 years before death and a Braak stage of IV or less. Cases were excluded if the following events were reported in the clinical reports: (i) history of a major depressive disorder severe enough to require psychiatric treatment; (ii) presence of a major neuropathological or inflammatory abnormality (e.g., cancer, bacterial infection); or (iii) evidence of a widespread disease process other than those under investigation (e.g., multiple sclerosis).

We focused on the dorsolateral prefrontal cortex (DLPFC) to investigate neuroinflammation. This is an area known to be affected in MDD and AD [17–19]. Particularly, DLPFC dysfunction is seen at early stage of AD [19,45], and is considered to be crucial due to its compensatory mechanism for neuropathological alteration [19]. Prefrontal cortex is also one of the brain areas which is most associated with MDD pathology [46] and both structural and functional abnormality are characterised in DLPFC of MDD patients [46–50].

### Ethical approval

Ethical approval for this work was provided by the Greater Manchester East NRES committee (ref 17/NW/0126). Informed written consent was given by the next of kin at the point of brain donation to the brain banks which supplied tissue for this study for the tissue to be used in research studies.

### Sample homogenisation

4°C RIPA lysis buffer (Merck) was combined with protease inhibitor (complete MINI protease inhibitor, Merck) and phosphatase inhibitor (phosSTOP, Merck) and added into 2ml homogenate tube containing fresh frozen brain tissue with 5-10 zirconia beads. All tubes were placed in a Precellys homogeniser for 2 x 15 sec at 6000 g. Then, tissue homogenates were centrifuged at 13,000 g for 15 minutes at 4°C. The supernatant was aliquoted into non-binding 96-well storage plates (Thermo Scientific) and total protein concentration was assessed using the Coomassie protein assay kit (Thermo Scientific). The storage plate was stored at -80°C until further analysis was conducted.

### ELISA

ICAM-1 and VCAM-2 were measured using ELISA to determine the permeability of blood brain barrier (R&D systems) as a marker of the downstream consequences of neuroinflammation. The samples were diluted in 1 in 200 for ICAM-1 and 1 in 80 for VCAM-1 before the ELISA to be conducted following the manufacturer’s protocol. The samples were measured in duplicate on each plate and the ELISA repeated twice. Expression of ICAM-1 and VCAM-1 was normalised against absolute protein level using Optima data analysis software (BMG Labtech). ELISA absorbance was measured at 450nm with the FLUOstar OPTIMA microplate reader (BMG Labtech).

### Multiplex assay

Inflammation associated marker proteins (IFN-γ, IL-1β, IL-2, IL-4, IL-6, IL-8, IL-10, IL-12p70, IL-13, and TNF-α) were measured by the V-Plex MesoScale Discovery (MSD) electrochemiluminescence multi-spot assay platform (MesoScale Diagnostics with the Proinflammatory Panel 1 Human Kit (cat. No. K15049D)). The samples were first diluted 1 in 2 and incubated overnight. Following this step, the rest of the analysis was conducted following the manufacturer’s protocol. The protein levels of inflammation associated marker were measured by Meso Quickplex SQ120 (MSD) and obtained values were normalised against total protein concentration (pg/mL/mg).

### Statistical analysis

The Shapiro-Wilk’s test was conducted to assess the normality of the data. If the data was normally distributed, linear regression was carried out including demographic variables as co-variates (i.e., age, sex, post-mortem delay and use of antidepressant if it is known). Those co-variates were included in the analysis as they may affect expression of neuroinflammatory markers independent from disease conditions. In addition to these, marital status, educational level and methods of suicide were included as co-variates for MDD and MDD control analysis. Suicidal methods were included as a co-variate as hanging (which is the most popular suicidal method) could potentially increase inflammation in the brain [51–53] and subsequently the level of cytokines and endothelial markers. Although we have included factors which could have affected inflammatory status independent from disease condition itself as much as possible, some factors were not included as data on these was not available, such as environmental conditions at the time of demise, smoking status, diet, or the presence of infections prior to death. We were able to include cause of death, life style (e.g., smoking and frequency of exercises) use of anti-inflammatory drugs or any other disease conditions (chronic inflammatory diseases). If the data were not normally distributed, Mann-Whitney U-test was conducted. A p <  0.05 was considered statistically significant. All values are shown as mean ±  standard deviation. Statistical analysis was conducted using JASP Team (Version 0.16. 3) and stata (version 17). The graphs created with GraphPad Prism 9.4. Statistical comparison was made in 2x2 design (MDD vs. MDD control; AD vs. AD control) to as control groups were diverse (e.g., age) to allow us to compare the group difference in more accurate manner (See S1 for 4x4 design (all cases of MDD, AD and controls).

## Results

### The MDD vs. MDD controls

#### The characteristics of the cohorts.

Please note that in the results the term MDD group includes both those with MDD and depressive disorder NOS. All MDD cases died by suicide, with most of them under psychotropic medications at the time of their demise, suggesting the presence of a severe depressive illness. Unfortunately, no clinical information was available on the number of depressive episodes experienced by individuals, nor the timing of depressive episodes in relation to the time of their demise. The cause of death implies that the patients were suffering with low mood at the time. As expected, there was a significant difference in cause of death (p < 0.001) as shown in the Table 1. Similarly to the cause of death, there was a significant difference in antidepressant medication use in last 3 months (p < 0.001) and in antipsychotic medication (p < 0.001) across the groups, with the MDD having the highest use, as expected (Table 1). Although there was overall significance in age (X^2^_3_ = 59.36, p < 0.001), there was no significant age difference between MDD and MDD controls. As expected, age of MDDs and MDD control groups were significantly different from early-stage AD and early-stage AD control groups (p < 0.001).

### Endothelial cell activation

**Fig 1:**
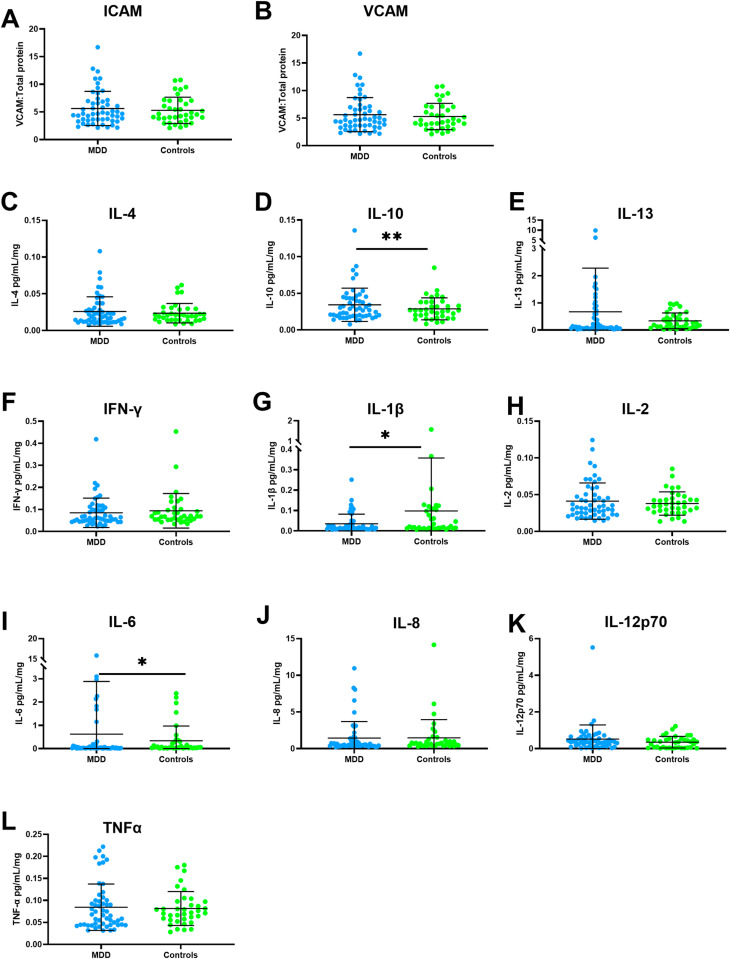
Expression level of endothelial activation markers and cytokine between MDD and control cohorts including endothelial activation (A) ICAM and (B) VCAM; anti-inflammatory markers (C) IL-4, (D) IL-10 and (E) IL-13; and pro-inflammatory markers (F) IFN-γ, (G) IL-1β, (H) IL-2, (I) IL-6, (J) IL-8 (K) IL-12p70 and (L) TNFα. p < 0.05 * , p < 0.01**.

Neither ICAM-1 (p = 0.998, beta = 0.000163, 95% Cl: -0.114 to 0.115) or VCAM-1 (p = 0.522, beta = -0.0476, 95% Cl: - 0.196 to 0.100) were significantly different between groups, indicating that depression was unlikely to affect endothelial cell activation in the DLPFC (Fig 1**A-B** ).

### Cytokine expression

A significant increase in the MDD *vs*. control groups was detected for IL-6 (MDD: 0.624 (2.262), controls: 0.338 (0.634); W = 1243, p = 0.032) and IL-10 (MDD: 0.0343 (0.0228), controls: 0.0288 (0.01500), p = 0.001, beta = 0.219, 95% CI: 0.0926 to 0.346). While IL-1β expression showed a significant decrease (MDD:0.035 (0.047), controls: 0.098 (0.26); W = 1225, p = 0.045) in the MDD cases. No difference was observed for the other cytokines (IL-4: W = 1014, p = 0.787, IL-13: W = 1112, p = 0.283, IFN-γ: W = 1086, p = 0.304, IL-2:p = 0.1, beta = 0.105, 95% Cl: -0.0205 to 0.231, IL-8: W = 1086, p = 0.389, IL-12p70: W = 813, p = 0.389, TNFα: W = 1045, p = 0.600) (Fig 1C-L).

### Early-stage AD vs early-stage AD controls

#### The characteristics of the cohorts.

All early-stage AD cases had neuropathological diagnosis of AD as stated in Table 1. As previously mentioned, the MDD cohorts differed in age compared to the early-stage AD cohorts (X^2^_3_ = 59.36, p < 0.001), meaning that early-stage AD cohort and their controls were approximately 35 years older in their age at death (mean age of 85 and 83.3 respectively) than MDD and their respective controls (mean age of 49.6 and 49.1). Neither age between early-stage AD and early-stage AD controls or sex ratio (p = 0.42) were significantly different.

### Endothelial cell activation

**Fig 2:**
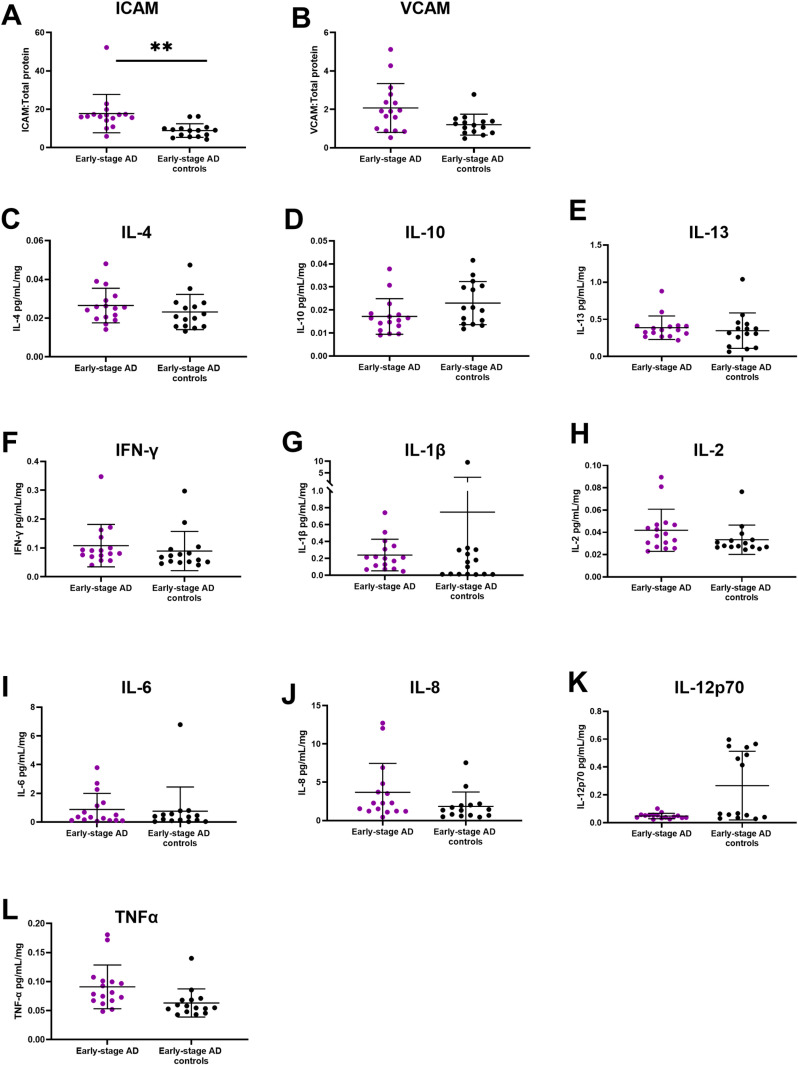
Expression level of endothelial activation markers and cytokine between early-stage AD and early-stage AD control cohorts including (A) ICAM and (B) VCAM; anti-inflammatory markers (C) IL-4, (D) IL-10 and (E) IL-13; and pro-inflammatory markers (F) IFN-γ, (G) IL-1β, (H) IL-2, (I) IL-6, (J) IL-8 (K) IL-12p70 and (L) TNFα. p < 0.05 * , p < 0.01**.

VCAM-1 did not show statistical difference between the 2 groups (early-stage AD: 2.071 (1.273), early-stage AD controls: 1.201 (0.544); p = 0.055, beta = -0.477, 95% CI: -0.966 to 0.012). Early-stage AD group (17.742 (9.997)) showed significantly higher ICAM-1 expression (p = 0.001** beta = -0.564, 95% CI: -0.873 to -0.255) compared to early-stage AD controls (8.877 (3.569)), indicating increased endothelial activation in the early stage of AD (Fig 2A-B).

### Cytokine expression

None of the cytokines were significantly different between early-stage AD and early-stage AD controls (IL-4: p = 0.994, beta = 0.001, 95% CI: -0.313 to 0.315, IL-10: p = 0.323, beta = 0.13, 95%CI: -0.139 to 0.405, IL-13: W = 141, p = 0.423, IFN-γ: p = 0.134, beta = 4.000, 95%CI: -1.323 to 9.33, IL-2: p = 0.245, beta = 4.443, 95%CI: -3.250 to 12.137, IL-8: p = 0.273, beta = -0.409, 95%CI: -1.162 to 0.343, IL-12p70: p = 0.498, beta = -2.601, 95%CI: -10.409 to 5.207, TNFα: p = 0.076, beta = -0.255, 95%CI: -0.539 to 0.029; Fig 2C-L)

## Discussion

Our study is the first post-mortem study investigating the potential neuroinflammatory link between depression and early-stage AD in the DLPFC. Both depression and AD are well associated with increased neuroinflammation, but this is traditionally assessed by biomarkers detected in CSF and serum or by positron emission tomography[54]. Very few studies have investigated the inflammatory brain environment in post-mortem human brain tissue. We observe that depression was not associated with changes in the inflammatory cytokine response as observed in early stage of AD with increased ICAM-1 expression. Instead, depression showed a different neuroinflammatory response with lower IL-1β and higher IL-6 and IL-10 expression compared to the controls. The inflammatory findings suggest that depression may have a different pattern of neuroinflammatory change to early-stage AD, implying that neuroinflammation is unlikely to be the mechanism by which depression might contribute to the development or progression of AD or at least not via a neuroinflammatory pathway similar to AD.

### Neuroinflammation in depression patients

Contradictory to many past studies, we showed a downregulation of IL-1β in depression patients. Although IL-1β is a proinflammatory cytokine and is considered to play a pivotal role in depression [55], increased inflammatory cytokines associated with depression is often derived from peripheral measure such as blood or CSF. The level of inflammatory cytokines found in these peripheral compartments does not always reflect the neuroinflammatory state in the brain. Interestingly, even in human blood, cumulative meta-analysis revealed that there is no consistent association with IL-1β and TNFα whilst IL-6 showed significance with depression in humans [56]. This means that despite meta-analyses reporting increases of peripheral IL-1β, TNFα and IL-6, only IL-6 levels reach statistical significance in the human brain. In fact, another human study using blood samples reported decreased IL-1β in middle aged male with depressive symptomatology[57]. IL-1β level in human CSF also showed inconsistent result with a meta-analysis describing one study with increased IL-1β in depression group while two studies showed no change in IL-1β level [58].

In contrast, level of IL-10 particularly in serum is generally increased with depression [59,60], which was also consistent with our findings. IL-10 is a primary anti-inflammatory cytokine and suggested to be increased in a response to the increase of IL-6 which is associated with suicide [61,62]. As IL-10 level is also known to be positively correlated with the severity of the disease [59], this may explain the increased IL-10 concentration found in depression brains compared to controls, similar to IL-6 in serum considering that all of the depression patients whose brains were used in this study committed suicide. Increased IL-10 may act as a compensatory mechanism against increased proinflammatory cytokines such as IL-6. However, IL-10 can also activate immune response in certain circumstances[61]. Furthermore, increased IL-10 level does not always reflect depression disease severity or suicide, suggesting that it may not always act as a compensatory mechanism. For example, a review article reported that suicidal attempt and suicidal idealisation did not affect IL-10 level in plasma [63]. Another study found that mRNA analysis in whole blood indicated that IL-10 was in fact decreased with suicidal idealisation and disease severity, while IL-6 level was increased [64]. These studies again show the complexity in immune response associated in depression. As far as we are aware, due to paucity of previous studies, there is no systematic review or meta-analysis conducted using post-mortem human brain studies of inflammatory cytokines in depression [58], which highlights the importance of our study. One previous study reported that expression of IL-1β was increased as well as IL-6 and TNF-α in PFC of the teenage suicide depression patients [65], consistent with peripheral findings. However, it remains unknown whether this also applies to older adults. Moreover, a rodent depression model, showed a lack of increased IL-1β but elevation of IL-6 [66], similar to our results in the human DLPFC, strongly indicating that studies are required to identify how IL-1β level changes in the brain with ageing and its subsequent effects. Although it remains controversial [58], increased activation of microglia (approximately 26%) in PFC has been shown in depression patients using positron emission tomography (PET) [67], implying that depression could be associated with chronic microglial activation. As IL-1β release can initiate microglia and astrocyte activation leading to the downstream synthesis of other proinflammatory cytokines in CNS[68,69] (e.g., IL-6), the chronic microglial activation reported in depression may activate a negative feedback to supress IL-1β. Consistent with other studies investigating peripheral inflammation, our study showed an increase of IL-6 in depression brains. IL-6 exhibits transient anti- and pro-inflammatory effects which are mainly determined by its receptor binding: proinflammatory effects are activated via IL-6 trans-signalling though the soluble IL-6 receptor (sIL-6R), while binding to membrane IL-6 receptor (IL-6R) exhibits anti-inflammatory effects[70,71]. Although previous studies have reported in depression, association with elevated peripheral inflammation [58] particularly IL-6 [64], whether IL-6 has pro- or anti-inflammatory effects in the disease remains unclear. An *In vitro* study using human hippocampal progenitor cells (HPC0A07/03C), showed that IL-6 inflammatory effect is concentration dependent as well as the concentration of IL-1β [72]. Decreased IL-1β and increased IL-6 may act as a compensatory mechanism in depression to counter act dysregulated neuroinflammatory response.

### Neuroinflammation associated with early-stage AD

Unlike depression, significant elevation of ICAM-1 was detected in our early-stage AD cohort. Although ICAM-1 is a vascular marker and not directly regarded as an inflammatory marker, it is a regulator of cellular response in inflammation and increase of its expression can be used as indicator of neuroinflammation in the brain [73]. Cell adhesion molecules (CAMs) elevation including ICAM-1 can be triggered by vascular deposition of amyloid β (Aβ), and CAMs could aid circulation of leukocytes [74]. Although the exact role of ICAM-1 in AD pathology remains unknown, ICAM-1 is found in the frontal cortex of AD patients and localised near Aβ plaques [75]. ICAM-1 forms extravascular aggregation [75,76], which may suggest an early defence mechanism against AD. In fact, increased ICAM-1 is observed in CSF across the different stages of AD including preclinical period, while markers of neuroinflammation such as IL-6 did not [77]. Moreover, an increase of ICAM-1 was observed to protect neurons from Aβ neurotoxicity in both *in vivo* and *in vitro* models and can improve cognitive deficit in Aβ-infused rat and 5xFAD mouse, further suggesting a potential protective role of ICAM-1 in early-stage AD pathology defence mechanism.

However, it is important to note that blood-brain barrier (BBB) failure, which is seen at the early stage of AD [78], can be caused by increased ICAM-1 expression [79]. Increased ICAM-1 in endothelial cells could lead to subsequent recruitment of inflammatory cytokines which again enhances neuroinflammation [79]. In fact, knockdown of ICAM-1 in human brain microvascular endothelial cells (HBMVECs) upregulated Aβ degradation by increasing neprilysin [80], while protective role of ICAM-1 was reported from AD model [81]. Although increased ICAM-1 associated with early-stage AD has been reported in various studies including ours, the role of ICAM-1 does not seem to be a simple driver for neuroinflammation, but seems to be specific the pathophysiomechanism of AD.

### Strengths and limitations

Unlike studies using peripheral measures of cytokines, our use of post-mortem brain tissue provides us with a more valid measure of neuroinflammatory status in the brain for both depression and AD. Our study uniquely addresses the neuroinflammatory status in a brain region known to be involved in the control of mood and affected in depression [82] It is possible that general increase in neuroinflammation was not observed in DLPFC at the early-stage AD group as none or only small amounts of neurofibrillary tangles are present at this stage. However, it is important to highlight that neuroinflammatory change observed in depression was not reflected in early-stage AD, despite the fact that general increase of inflammation is reported in both diseases. Future studiesshould examine brain areas which would have relatively advanced neurofibrillary tangles such as the medial temporal cortex area, which would improve our understanding of the role of neuroinflammation. It would also be helpful to examine brain areas with shared influence from AD and depression such as the limbic system, but tissue was not available from these brain areas for this study.

We controlled for co-variates as much as possible, but there are limitations such as age of each cohort and limited clinical information. Indeed, no information was available on whether the individuals in the depression group and their controls were taking anti-inflammatory medications or whether co-morbid inflammatory illnesses were present, which could have affected the inflammatory status measured in the brain. Finally, it is possible that the cytokines could have been affected by post-mortem delay, although we controlled for this as far as we were able to.

Although there were 35 years of mean age difference between depression and early-stage AD groups, which could have influenced the baseline neuroinflammation, none of the inflammatory markers were different between early-stage AD control and depression control (Supplement 1), indicating that age does not seem to play a major role in the neuroinflammatory status. ICAM-1 was the only marker which early-stage AD control had significantly more than depression control, showing that ICAM-1 level in the DLPFC is increased with age, which is also consistently with the previous finding in the orbitofrontal cortex [83]. Although increase of ICAM-1 could lead to further recruitment of inflammatory cytokines which can result in an increase inflammation [79], such an effect was not seen in our study. Indeed, increased baseline neuroinflammation has been suggested in many studies, but age-related differences in cytokines are often reported using rodent models and also increased neuroinflammation is most pronounced in the hippocampus compared to the other brain regions [84]. These suggest that age-related neuroinflammatory could be negligible in the DLPFC of human brains and it is likely to be affected by disease conditions.

It is important to note that all of the brains from depression cases were obtained from people who had died by suicide, which strongly indicates the results of severe depression and therefore the findings in this study may only reflect severe depression cases. This could explain the inconsistency of the finding in this study compared to others as mentioned previously. However, it is crucial to highlight that most of past studies have only examined peripheral samples (i.e., CSF and blood), and therefore our finding is still useful. In addition to this, restricted sample sizes may contribute the limited findings.

## Conclusions

Our hypothesis that depression and early-stage AD share common neuroinflammatory characteristics was not validated by our findings, suggesting that depression might not increase AD risk via the same neuroinflammatory pathway. However, the increased neuroinflammation reported in the early stage of AD might be induced by BBB disruption whilst the neuroinflammation associated with depression appear to independent from BBB changes. It is important to identify specific neuroinflammatory pathways associated with each disease and to determine downstream effects of neuroimmune dysregulation. Future studies should include larger numbers of individuals and tissue from a greater number of brain areas in order to characterise in-depth the neuroinflammatory milieu in depression.

## Supporting information

S1 Expression level of endothelial activation markers and cytokine between early-stage AD, early-stage AD control, MDD and MDD control cohorts across 4 groups.
(DOCX)
