# Correction: Depression as a risk factor for Alzheimer’s disease: A human post-mortem study

**DOI:** 10.1371/journal.pone.0340296

**Published:** 2026-01-05

**Authors:** Mizuki Morisaki, Farnoosh Rezaali, Laurie C. Lau, Delphine Boche, Golam M. Khandaker, Gustavo Turecki, Lindsey I. Sinclair

The Funding statement for this article is incorrect. The correct Funding statement is as follows: We are grateful to the Alzheimer’s Society (grant 518) and the Elizabeth Blackwell Institute at the University of Bristol and the Wellcome trust institutional strategic support fund (204813/Z/16/Z) who supported Dr. Sinclair and Dr. Morisaki during this research project. Prof. Khandaker is supported by the Wellcome Trust (Grant No. 201486/B/16/Z), the Medical Research Council, UK (Grant No. MC_UU_00011/1); Grant No. MR/S037675/1; and Grant No. MR/W014416/1), and the National Institute of Health Research Bristol Biomedical Research Centre, UK (Grant No. NIHR203315). The South West Dementia Brain Bank is funded by BRACE, the Alzheimer’s Society & Alzheimer’s Research UK as part of Brains for Dementia Research. The Douglas Bell Brain Bank is funded by the fonds du recherche du Quebec-Santé through the Réseau québécois sur le suicide, les troubles de l’humeur et les troubles associés, as well as Brain Canada Foundation. Parts of this work contributed to an MSc in neuroscience for Miss Rezali. The funders had no role in study design, data collection and analysis, decision to publish, or preparation of the manuscript.
