# Supplementary material for: Depression as a risk factor for Alzheimer’s disease: A human post-mortem study
Source: PLoS One. 2025 Apr 3;20(4):e0320561. doi: 10.1371/journal.pone.0320561 (PMC11967934; doi:10.1371/journal.pone.0320561)
Supplement: S1 — Expression level of endothelial activation markers and cytokine between early-stage AD, early-stage AD control, MDD and MDD control cohorts across 4 groups. (DOCX) [file pone.0320561.s001.docx]

**Supplement 1: Expression level of endothelial activation markers and cytokine between early AD, early AD control, MDD and MDD control cohorts across 4 groups**

**
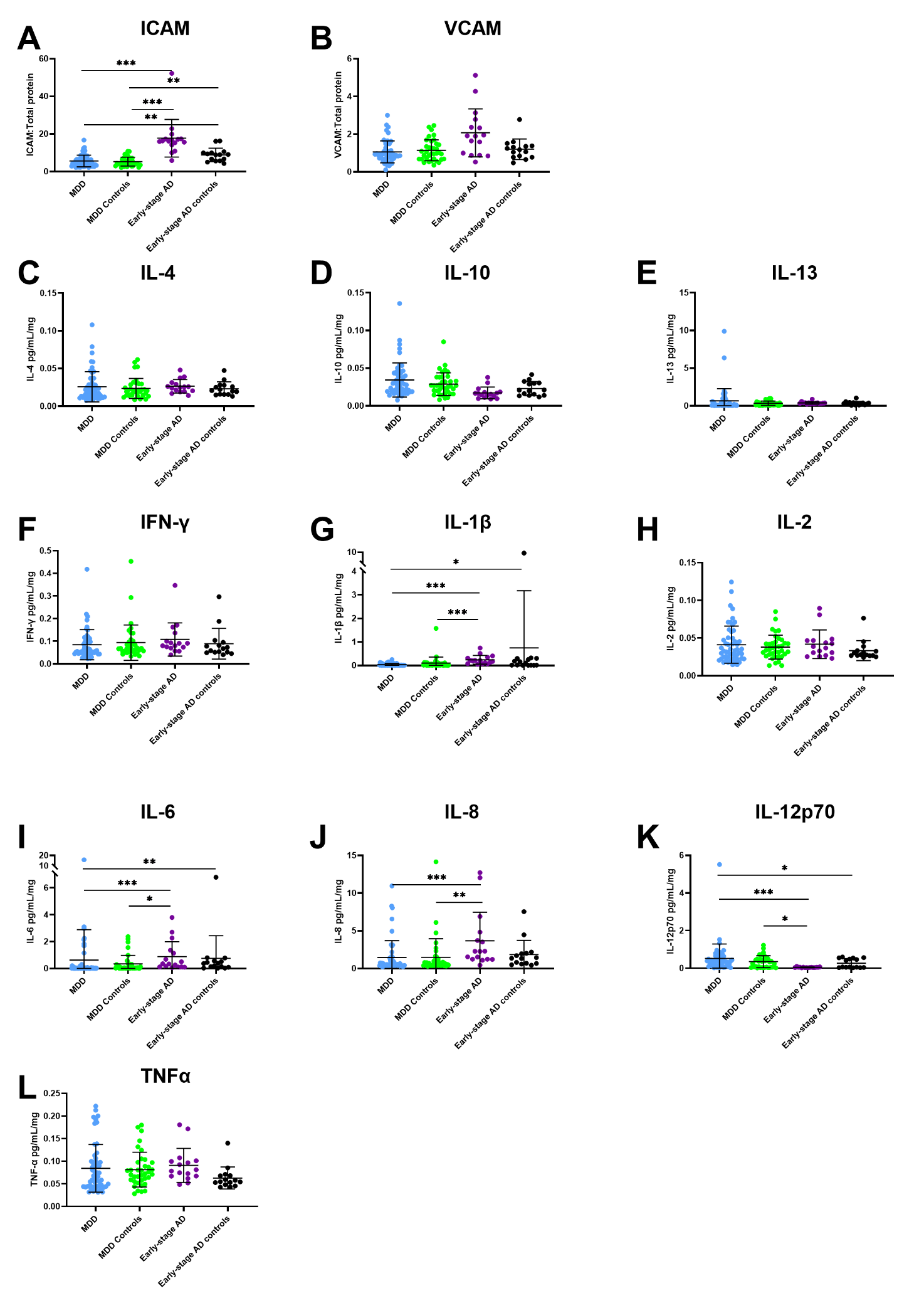
**

**Supplement figure 1: Expression level of endothelial activation markers and cytokine between early AD, early AD control, MDD and MDD control cohorts** including (A) ICAM and (B) VCAM; anti-inflammatory markers (C) IL-4, (D) IL-10 and (E) IL-13; and pro-inflammatory markers (F) IFN-γ, (G) IL-1β, (H) IL-2, (I) IL-6, (J) IL-8 (K) IL-12p70 and (L) TNFα. p<0.05*, p<0.01**, p<0.001***.

**Methods**

Four groups (MDD, MDD control, early AD and early AD control) were compared using ANCOVA for normally distributed data and Kruskal Wallis for those which were not. Co-variates were included in ANCOVA (i.e. age, sex, post-mortem delay and use of antidepressant if it is known). If there is any statistical significance, post-hoc tests were conducted (Bonferroni test for ANCOVA and Dunn’s with Bonferroni correction for Kruskal-Wallis test).

**Results**

*Endothelial cell activation*

Expression of VCAM was similar across the groups (F_3,111_=1.787, p=0.154), but ICAM expression was significantly different among groups (X^2^(3)=45.306, p<0.001): early AD group showed higher expression compared to MDD (p<0.001) and MMD control (p<0.001). Early AD control group also showed higher ICAM expression than MDD and MDD control, suggesting that ICAM expression was overall higher in AD cohorts compared to MDD cohort and also possible age effect in its expression change.

*Cytokine expression*

Level of expression in anti-inflammatory cytokines were the same across the groups (IL-4: X^2^(3)=3.836, p=0.28, IL-10: F_3,113_=0.778, p=0.155, and IL-13: X^2^(3)=6.129, p=0.105). Similarly, three pro-inflammatory associated cytokines expression were the same across the groups (IFN-γ: X^2^(3)=4.761, p=0.19, IL-2: F_3,113_=0.943, p=0.422 and TNFα: X^2^(3)=5.854, p=0.119).

However, there are several pro-inflammatory cytokines showed significant expression difference among four groups. For instance, IL-1β expression (X_2_(3)=31.959, p<0.001). IL-1β expression was higher in early AD compared to MDD (p<0.001) and MDD control (p<0.001). IL-1β expression was also higher in early AD control compared to MDD (p=0.042). Similar expression patterns were shown in IL-6 (X^2^(3)=25.592, p<0.001): early AD showed higher expression than MDD (p<0.001) and MDD control (p=0.026). AD control also showed higher IL-6 expression compared to MDD (p=0.004). In IL-8 (X^2^(3)=21.088, p<0.001), early AD had higher expression than MDD (p<0.001) and MDD control (p=0.003).

IL-12p70 (F_3,113_=7.327, p<0.001), MDD had more IL-12p70 expressed than early AD (p<0.001) and early AD control (p=0.032). MDD control also had higher IL-12p70 than early AD.

**Summary**

This is as an alternative statistical analysis of our findings. We have analysed early AD and MDD cohorts separately and compared them to their own control as we were interested whether inflammatory response changes seen in MDD are similar to the ones seen in early AD and also age differences in the controls (i.e. 8. In this analysis, we have compared four groups together and investigated whether there are any differences between MDD and early AD conditions including potential age effect.

In terms of endothelial activation, higher ICAM expression was observed in early AD compared to MDD and MDD control, but its expression was not significantly higher than its own control, which was different from our finding on the main article. There may be an age effect in ICAM expression as early AD control showed significantly higher ICAM expression compared to MDD control. ICAM expression may increase with age as mean age for early AD cohorts (mean age for both early AD and early AD control) was approximately 35 years older than MDD cohorts (MDD and MDD control).

Most of pro-inflammatory cytokines, which had significant difference (i.e. IL-6, IL-1β and IL-8), were expressed at higher level in early AD group compared to the rest excluding IL-12p70. IL-12p70 showed the opposite pattern, meaning that its expression was higher in MDD group compared to early AD group.
